# Does an Insect's Unconditioned Response to Sucrose Reveal Expectations of Reward?

**DOI:** 10.1371/journal.pone.0002810

**Published:** 2008-07-30

**Authors:** Mariana Gil, Randolf Menzel, Rodrigo J. De Marco

**Affiliations:** Free University of Berlin, Department of Biology/Chemistry/Pharmacy, Institute of Biology/ Neurobiology, Berlin, Germany; University of Sydney, Australia

## Abstract

We asked whether and how a sequence of a honeybee's experience with different reward magnitudes changes its subsequent unconditioned proboscis extension response (PER) to sucrose stimulation of the antennae, 24 hours after training, in the absence of reward, and under otherwise similar circumstances. We found that the bees that had experienced an increasing reward schedule extended their probosces earlier and during longer periods in comparison to bees that had experienced either decreasing or constant reward schedules, and that these effects at a later time depend upon the activation of memories formed on the basis of a specific property of the experienced reward, namely, that its magnitude increased over time. An anticipatory response to reward is typically thought of as being rooted in a subject's expectations of reward. Therefore our results make us wonder to what extent a long-term ‘anticipatory’ adjustment of a honeybee's PER is based upon an expectation of reward. Further experiments will aim to elucidate the neural substrates underlying reward anticipation in harnessed honeybees.

## Introduction

Recently [1], we trained honeybees to associate colours with sucrose reward in a setting closely resembling a natural foraging situation, and tested whether their sequence of experience with different volumes of sugar solution changed their subsequent foraging behaviour in the absence of reward and under otherwise similar circumstances. We found that those bees that had experienced increasing volumes of sugar reward during training assigned more time to flower inspection when tested 24 and 48 hours after training. These animals behaved differently neither because they were fed more or faster nor because they had more strongly associated the related predicting signals, thereby indicating that bees can develop long-term expectations of reward, in that their behaviour in the absence of reinforcement can be the subject of changes at a later time on the basis of a specific property of an experienced reward, namely, that its magnitude increased over time. Indeed, the term ‘reward expectation’ [2], [3] refers to behavioural adaptations that depend upon the formation and subsequent activation of memories about specific properties of a given reward, whose recollection is eventually triggered in the absence of reinforcement by the cues and events predicting such a reward. Eventually, an utterly important first step to elucidate the neural mechanisms underlying such a form of learning is to develop a laboratory procedure suitable to examine behavioural adaptations depending on memories of specific reward properties. This would allow experiments based on pharmacological and electrophysiological approaches. We took advantage of the honeybees' proboscis extension response, or PER [4], [5], in order to develop such a procedure.

A honeybee's PER possesses at least two features indicating that it might constitute a suitable behavioural response to reveal memories about specific reward properties in the laboratory. First, PER in non-satiated honeybees is reflexively elicited when chemoreceptors in the animals' antennae, proboscis and tarsi are stimulated with sucrose [5]. Sugar solution is a honeybee's primary source of energy, and sucrose thus acts as an appetitive stimulus; this reflects response specificity. Second, a PER's motor program consists of at least three phases: extension, repeated licking and retraction [6]. These three phases have different thresholds and require integration of internal state conditions, evaluation of external stimuli, and muscle coordination. The variability of the temporal pattern and the strength of the motor response, in relation to both the nature of the stimulus that releases it and the subject's experience with such stimulus, have been described elsewhere [6]–[8]. What is important here is that a honeybee's PER is a rather flexible -unconditioned- response whose innately defined parameters can subsequently be calibrated through learning. Based on these two features, response specificity and behavioural flexibility, we benefited from an experimental design analogous to that of our experiments with free-flying bees [1], and asked whether and how a sequence of a honeybee's experience with different reward magnitudes changes its subsequent proboscis extension response to sucrose stimulation of the antennae, in the absence of reward and under otherwise similar circumstances.

## Methods

To this end, we caught honeybees (*Apis mellifera carnica*) at a hive's entrance and harnessed them in metal tubes by strips of tape between their head and thorax, so that they could freely move their antennae and mouthparts. After harnessing, we placed the bees in racks, fed them with 10 µl of 1.2 M sucrose solution, and kept them overnight in a dark humidified chamber. We presented the bees with three ‘training’ trials during the next morning. In the study of associative learning in honeybees, the term ‘training’ trial often refers to a CS-US presentation; here, however, it specifically refers to the sucrose stimulation of an animal's antenna and the subsequent presentation of a given volume of sugar solution to its proboscis. Such a distinction is important because our analysis focused on a honeybee's PER as an ‘unconditioned’ response to sucrose stimulation of the antenna. While the inter-trial interval lasted 10 minutes, each training trial lasted approximately 30 s. Removing a bee from a rack to the training site was followed by a 10 s accommodation period, after which we first stimulated one of its antennae for 2 s by touching it with a toothpick soaked in an unscented, 1 M sucrose solution, and then fed the animal for 10 s with a given volume of the same sucrose solution delivered to its proboscis by means of a micrometer syringe. After the 10 s feeding period, the bee remained in the training site for 7 s, and was then placed back in the rack. In order to leave aside possible side-specific effects of sucrose stimulation of the antenna on the development of memories about specific reward properties, we always presented only one, either left or right, of an animal's antennae with sucrose solution during both training and testing.

We performed two variable and three constant experimental series. They differed in the volume of sucrose solution that the bees received throughout the three consecutive training trials. In the variable series, we offered either increasing (small-medium-large) or decreasing (large-medium-small) volumes of sugar solution throughout the three training trials. The bees in the increasing series received 0.4 µl, 1 µl and 1.6 µl, while the bees in the decreasing series received 1.6 µl, 1 µl and 0.4 µl in the first, second and third trial, respectively. Both series thus offered the same volume of sugar solution during training. In the constant series, we offered the same volume of sugar solution (small, medium or large) during the three successive training trials, and the bees of the ‘small’, ‘medium’ and ‘large’ series received 0.4 µl, 1 µl and 1.6 µl of sugar solution per trial, respectively. The evening following training, bees were fed with 5 µl of 1.2 M sucrose solution, and kept overnight inside a dark humidified chamber. To feed the bees after both harnessing and training, we released their PERs by stimulating their proboscis with sugar solution, instead of their antennae, thereby avoiding triggering PERs in a way similar to that of training. We tested the animals 24 h after training. Testing consisted of a 10 s accommodation period followed by a 2 s stimulation of the antenna similar to that of training. During testing, we video-recorded the animals' proboscis extension responses at 30 frames s^−1^. Subsequently, we quantified measures arising from the animals' responses to sucrose stimulation by analyzing the videos frame by frame. Bees that did not respond to sucrose stimulation during training were excluded from the analysis. We focused on several parameters related to the animals' PER's motor program. Thus, we measured a PER's ‘reaction-time’ (in ms), as the time elapsed between the onset of sucrose stimulation of the antenna and the first movement of the proboscis, provided that such movement subsequently led to a successful extension of the animal's proboscis, scored as such if the proboscis crossed an imaginary line between the tips of the opened mandibles. We also estimated a PER's strength by measuring: 1) the number of times that a bee extended its proboscis during testing, or ‘#PE’, 2) the mean duration of the proboscis extension, or ‘mean PE’, 3) the cumulative duration of the proboscis extension, or ‘CPE’, 4) the number of licking events, or ‘#L’, as the number of exposures of the animal's glossa, 5) the mean duration of licking, or ‘mean L’, and, finally, 6) the cumulative duration of licking, or ‘CL’. It has been reported that bees may differ with respect to their responsiveness to sucrose solution [9], and that such responsiveness may influence how well a bee can learn and remember tactile stimuli [10]. Before training, therefore, we tested the bees for their spontaneous responsiveness to sugar solutions of different sucrose concentrations, and then assigned the subjects to the different experimental series so that each series involved a similar proportion of bees from the different sucrose responsiveness categories previously defined. Later on, however, we pooled data from animals with different sucrose responsiveness, simply because their performance during both training and testing was invariant to such responsiveness (data not shown).

Data did not fulfil the requirements of parametric tests and were then analyzed by means of Kruskal-Wallis tests, Dunn's multiple comparison, and Bartlett test (with the corresponding alpha level adjustment).

## Results

**Figure 1:**
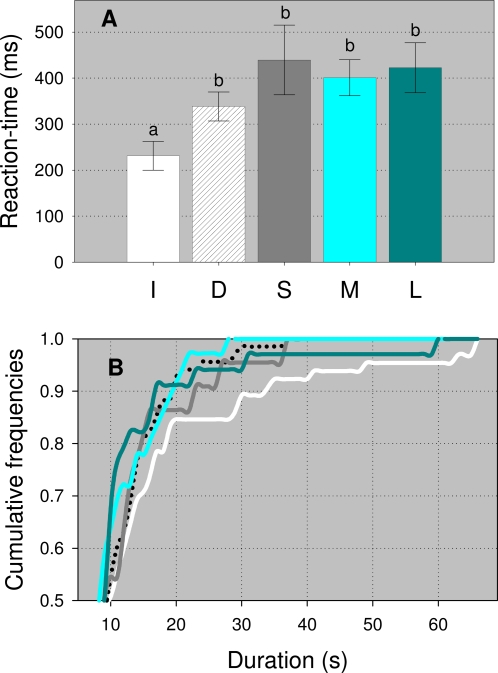
Reaction times and CPE durations. A) Mean (±s.e.m) reaction-time (in ms) of the animals' proboscis extension responses. B) Cumulative frequencies of CPE durations (in seconds). The data from the different series are shown separately: white, dashed, gray, cyan and dark-cyan bars and lines correspond to the increasing (I, n = 63), decreasing (D, n = 68), small (S, n = 22), medium (M, n = 35) and large (L, n = 34) series, respectively. In A, different letters indicate statistical differences across series: Kruskal-Wallis test, H = 26.66, P<0.001; Dunn's multiple comparisons P<0.001.

All the bees extended their probosces successfully during the experiments. We found a significantly shorter reaction-time in the bees of the increasing series, in comparison to that of the bees of the decreasing and the constant series (Fig 1A). Moreover, an analysis of the cumulative frequencies of the ‘CPE’ durations from the different series showed that the bees of the increasing series were more likely to extend their proboscis during longer periods, in comparison to the bees of the remaining series (Fig 1B). Thus, ‘CPE’ had a higher variance in the increasing series than in the decreasing, small and medium series, and such variance did not change across the constant series (Bartlett test, P _I vs D_<0.0001, P _I vs S_ = 0.002, P _I vs M_<0.0001, P _I vs L_ = 0.02, P _D vs S_ = 0.1, P _D vs M_ = 0.3, P _D vs L_ = 0.0009, P _S vs M_ = 0.06, P _S vs L_ = 0.1, P _M vs L_ = 0.001; differences should be taken as significant only if P<0.005). The mean values of ‘#PE’, ‘mean PE’, ‘CPE’, ‘#L’, ‘mean L’, and ‘CL’ did not change across series (Table 1).

**Table 1:** Mean values (±s.e.m.) of variables characterizing a PER's strength.

	Variable series		Constant series			Kruskal-Wallis test
	Increasing	Decreasing	Small	Medium	Large	
**#PE**	1.9±0.2	1.5±0.1	1.7±0.2	1.8±0.2	1.8±0.2	H = 0.7, P = 0.9
**Mean PE (s)**	9.9±1.5	8.3±0.8	7.9±1.6	6.8±0.8	7.8±1.7	H = 2.9, P = 0.5
**CPE (s)**	14.6±1.8	10.9±0.8	11.2±1.7	9.9±1.0	10.7±1.8	H = 2.2, P = 0.7
**#L**	7.4±1.8	8.2±1.3	4.5±2.1	5.3±1.0	7.2±2.9	H = 3.3, P = 0.5
**Mean L (ms)**	373.6±31.9	377.1±25.7	445.1±44.4	359.1±18.5	322.1±28.5	H = 7.0, P = 0.1
**CL (s)**	2.7±0.7	2.9±0.5	2.0±0.8	2.0±0.4	3.6±2.1	H = 3.4, P = 0.5

## Discussion

We found that the bees that had experienced an increasing reward schedule extended their probosces earlier and during longer periods in comparison to the bees that had experienced either decreasing or constant reward schedules (Fig 1A, B). The different performance of the bees of the increasing and decreasing series cannot be accounted for by assuming that their behaviour during testing reflects their most recent reward experience. By this argument, the bees of the decreasing series might only retain information on the small volume, and the bees of the increasing series might only retain information on the large volume; next, their behavior during testing should be controlled by this information. If this were the case, similar results must be expected between the large and the increasing series, and between the small and decreasing series, as well as differences among the constant series. Nevertheless, we found differences in the reaction-time between the animals of the increasing and the large series, and neither the reaction-time nor the CPE changed across the constant series (Fig. 1A, B). Similarly, our results cannot be explained on the basis of the total amount of reward that the bees received during training. If this were the case, the bees of the constant series should have behaved differently during testing, because they had attained different volumes of sugar solution during training, and the bees of the increasing and decreasing series should have behaved similarly during testing, because they had attained similar volumes of solution during training. Clearly, this has not been the case (Fig. 1A, B). In principle, multiple exposures to sucrose might provide an opportunity for habituation to such a stimulus. Therefore the increasing series could eventually be interpreted as less affected by habituation than the other series. However, the differences that we found among the several experimental series can not be explained in this way, simply because habituation of the sucrose response in bees requires tens of stimulation repetitions [11]. Taken together, therefore, our results unambiguously document that an increasing reward schedule has long-term effects on the ‘eagerness’ and the ‘strength’ of a honeybee's proboscis extension response to sucrose stimulation of the antennae, and indicate that these effects at a later time depend upon the activation of memories formed on the basis of a specific property of the experienced reward, namely, that its magnitude increased over time.

These results resemble our findings with free-flying bees [1], in that specific long-term reward memories can lead to later behavioural adaptations in the absence of reinforcement. In principle, our experimental design might have also allowed us to reveal specific reward memories arising from a decreasing reward schedule. If the effects of such memories on a bee's PER to sucrose stimulation were symmetrically opposite to those of the memories arising from an increasing reward schedule, then the bees exposed to a decreasing reward schedule would have shown longer reaction times and shorter PE durations, in comparison to measures from the bees that had been exposed to either increasing or constant reward schedules. Our results do not support this view, however, since we found no difference among the subjects of the decreasing and the constant groups. Since all the bees included in the present analysis successfully extended their probosces during testing, one possible explanation for such a lack of differences is that the system controlling both the reaction-time and duration of a honeybee's PER is much more sensitive to positive than to negative changes in reward magnitude. If this were the case, using larger differences in reward magnitude would be useful to reveal possible effects of a decreasing reward schedule on a honeybee's PER. In our experiments with free-flying bees [1], it was also an increasing reward schedule during training, and not a decreasing one, that had long term effects on the bees' subsequent behaviour during testing. Yet, because non-satiated bees extend their probosces reflexively in response to sucrose stimulation of the antenna, it might well have happened that a form of ceiling effect prevented us from detecting the effects of a decreasing reward schedule on a honeybee's PER. Characterizing the PERs of untrained honeybees would prove helpful to distinguish among these and other hypotheses. Eventually, it would also be interesting to examine whether and how a PER's reaction-time changes during training, and how the magnitude and frequency of reward variations relate to the adjustment a honeybee's PER.

Our procedure can be improved by increasing the spatial and temporal precision of the sucrose stimulation of the antenna. A substitution of the movements of the proboscis by the activity of a muscle responsible for such movements, called M17 e.g., [6]–[8], would also prove fruitful for further analyses of the neural substrates underlying long-term adjustments of a honeybee's PER. Recently, we have revealed comparable short-term adjustments, as well as their associated form of extinction throughout a series of unrewarding trials, by using electrophysiological recordings of M17 activity coupled to video recordings. This is important because honeybees allow recording neuronal activity over long periods of time e.g., [12], making it possible to trace the neural substrates of learning related plasticity. Moreover, global and local injections of pharmaca into a honeybee's brain allow manipulating transmitter and modulator systems [13]. This would help in characterizing the circuitry underlying a form of reward anticipation as revealed in the present context. Interestingly, elements of the pathway mediating a PE's response to sucrose have already been identified [14]–[16], and the same holds true for its modulatory actions on additional pathways [17]–[19]. Evidence supports the view that neurons of the VUM system of the suboesophageal ganglion [19] encode the reinforcing function of sucrose reward in olfactory conditioning [13], [18], [20]–[22], and it will be a task for future research to record and pharmacologically manipulate such neurons in order to search for neural correlates of reward memory. In addition, our experiments with free-flying bees showed that reward memories arising from increasing reward schedules are independent of classical and/or operant associations between an initially meaningless visual stimulus and the offered reward [1]. Further experiments using conditioning of a honeybee's PER [4], [5], [23]–[25] and reinforcing schedules of variable reward magnitudes would help to elucidate whether and to what extent an increasing reward schedule would influence a conditioned PE response. Moreover, Pavlovian conditioning does not require that the CS be initially neutral. It is a matter of experimental convenience that one usually uses a stimulus that does not elicit any unconditioned response because this makes it easier to demonstrate emergence of the CR to that CS. Hence, we might ask whether the application of sucrose solution on a honeybee's antennae could also serve as a CS for subsequent reward. In fact, water vapour emanating from a drop of sucrose solution may reach the antennae immediately before sucrose stimulation, and water vapour is known to act as a CS. In the present context, such a form of CS/US conditioning would have happened in all of our experimental groups, and there is no reason why the increasing group should have associated the CS component of sucrose more strongly than the other groups. Still, it will be a task for future research to study the potential effect of Pavlovian conditioning on increasing reward schedules, and vice versa.

Apparently, animals assign rewards with ‘motivational values’ [26] depending on the probability, quality and quantity of such reward. It is said that varying a reward's subjective value can lead to the adjustment of a subject's anticipatory response to such a reward. The adjusted response is, in addition, typically thought of as being rooted in the subject's already developed expectation of reward [26]. We suggest that when a harnessed bee extends its proboscis reflexively in response to sucrose stimulation of the antenna and receives either variable or constant volumes of sucrose solution throughout several trials, a built-in ‘change detector’ computes the difference in volume across trials. An internal estimate of an expected reward follows the detection of changes in reward magnitude. Such estimate is then combined with additional inputs determining a subjective evaluation of reward, and, finally, a ‘motivational value’ arises from such evaluation. Next, a reward of increasing magnitude is assigned with a high motivational value, and this leads, in turn, to the adjustment of the animal's PER. Expectations of reward are thought to be part and parcel of a set of rules controlling goal-seeking behaviours, and one should ask to what extent a long-term anticipatory adjustment of a honeybee's PER is rooted in a form of expectation of reward. Honeybees already proved fruitful to study how brain connectivity is eventually mapped to behaviour e.g., [27], [28], meaning that, if that were the case, a rather simple unconditioned response would help to identify, and eventually also to characterize, the neural correlates of such a form of learning in the honeybee brain.
